# MUC4 and MUC1 Expression in Adenocarcinoma of the Stomach Correlates with Vessel Invasion and Lymph Node Metastasis: An Immunohistochemical Study of Early Gastric Cancer

**DOI:** 10.1371/journal.pone.0049251

**Published:** 2012-11-13

**Authors:** Yukihiro Tamura, Michiyo Higashi, Sho Kitamoto, Seiya Yokoyama, Masahiko Osako, Michiko Horinouchi, Takeshi Shimizu, Mineo Tabata, Surinder K. Batra, Masamichi Goto, Suguru Yonezawa

**Affiliations:** 1 Department of Human Pathology, Field of Oncology, Kagoshima University Graduate School of Medical and Dental Sciences, Sakuragaoka, Kagoshima, Japan; 2 Department of Internal Medicine, Ohsumi-Kanoya Hospital, Kanoya, Kagoshima, Japan; 3 Department of Surgery, Kagoshima-shi Medical Association Hospital, Kagoshima, Japan; 4 Department of Pathology, Kagoshima-shi Medical Association Hospital, Kagoshima, Japan; 5 Departments of Biochemistry and Molecular Biology, Eppley Institute for Research in Cancer and Allied Diseases, University of Nebraska Medical Center, Omaha, Nebraska, United States of America; 6 National Sanatorium Hoshizuka-Keiaien, Kanoya, Kagoshima, Japan; Wayne State University School of Medicine, United States of America

## Abstract

We have previously reported that MUC4 expression is a poor prognostic factor in various carcinomas. Our previous study also showed that MUC1 expression in gastric cancers, including the early and advanced stages is a poor prognostic factor. In the present study, the expression profiles of MUC4 and MUC1 were examined by immunohistochemistry (IHC) using two anti-MUC4 monoclonal antibodies (MAbs), 8G7 and 1G8, and anti-MUC1 MAb DF3 in 104 gastrectomy specimens of early gastric adenocarcinoma with submucosal invasion (pT1b2), including 197 histological subtype lesions. Before the IHC study of the human specimens, we evaluated the specificity of the two MAbs by Western blotting and IHC of two MUC4 mRNA expressing gastric cancer cell lines. MAb 8G7 reacted clearly, whereas MAb 1G8 did not show any reactivity, in either Western blotting or IHC. In the IHC of the gastric cancers, the expression rates of MUC4/8G7 detected by MAb 8G7, MUC4/1G8 detected by MAb 1G8 and MUC1/DF3 detected by MAb DF3 in well differentiated types (70%, 38/54; 67%, 36/54; 52%, 28/54) were significantly higher than those in poorly differentiated types (18%, 10/55; 36%, 20/55; 13%, 7/55) (*P*<0.0001; *P* = 0.0021; *P*<0.0001), respectively. The MUC4/8G7 expression was related with lymphatic invasion (r = 0.304, *P* = 0.033). On the other hand, the MUC4/1G8 expression was related with lymphatic invasion (r = 0.395, *P* = 0.001) and lymph node metastasis (r = 0.296, *P* = 0.045). The MUC1/DF3 expression was related with lymphatic invasion (r = 0.357, *P* = 0.032) and venous invasion (r = 0.377, *P* = 0.024). In conclusion, the expression of MUC4 as well as MUC1 in early gastric cancers is a useful marker to predict poor prognostic factors related with vessel invasion.

## Introduction

Gastric cancer is the fourth most common cancer worldwide and more than 90% of gastric cancers are adenocarcinomas [1]. Recently, in Japan, early detection by the routine endoscopic examination in the gastroenterology clinics has resulted accurate diagnoses and effective surgical or endoscopic treatments, resulting in a relatively better prognosis. In the analysis of 11,261 patients with gastric cancer treated by gastric resection, the TNM 5-year survival rate for stage IA was 91.8% and for stage IB the survival rate was 84.6% [2]. For the early gastric cancers, an endoscopic submucosal dissection (ESD) is the first choice treatment in Japan, but the criteria of the additional surgery including lymph node dissection after the ESD are still controversial [3].

Our series of immunohistochemistry (IHC) studies for mucin expression in various human neoplasms have demonstrated that the expression of the MUC1 mucin (pan-epithelial membrane-associated mucin) is related with invasive proliferation of the tumors and poor outcome of the patients, whereas the expression of the MUC2 mucin (intestinal type secretory mucin) is related with the non-invasive proliferation of the tumors and a favorable outcome for the patients [4], [5]. Our previous study showed that MUC1 expression in gastric cancers is a poor prognostic factor [6].

**Figure 1:**
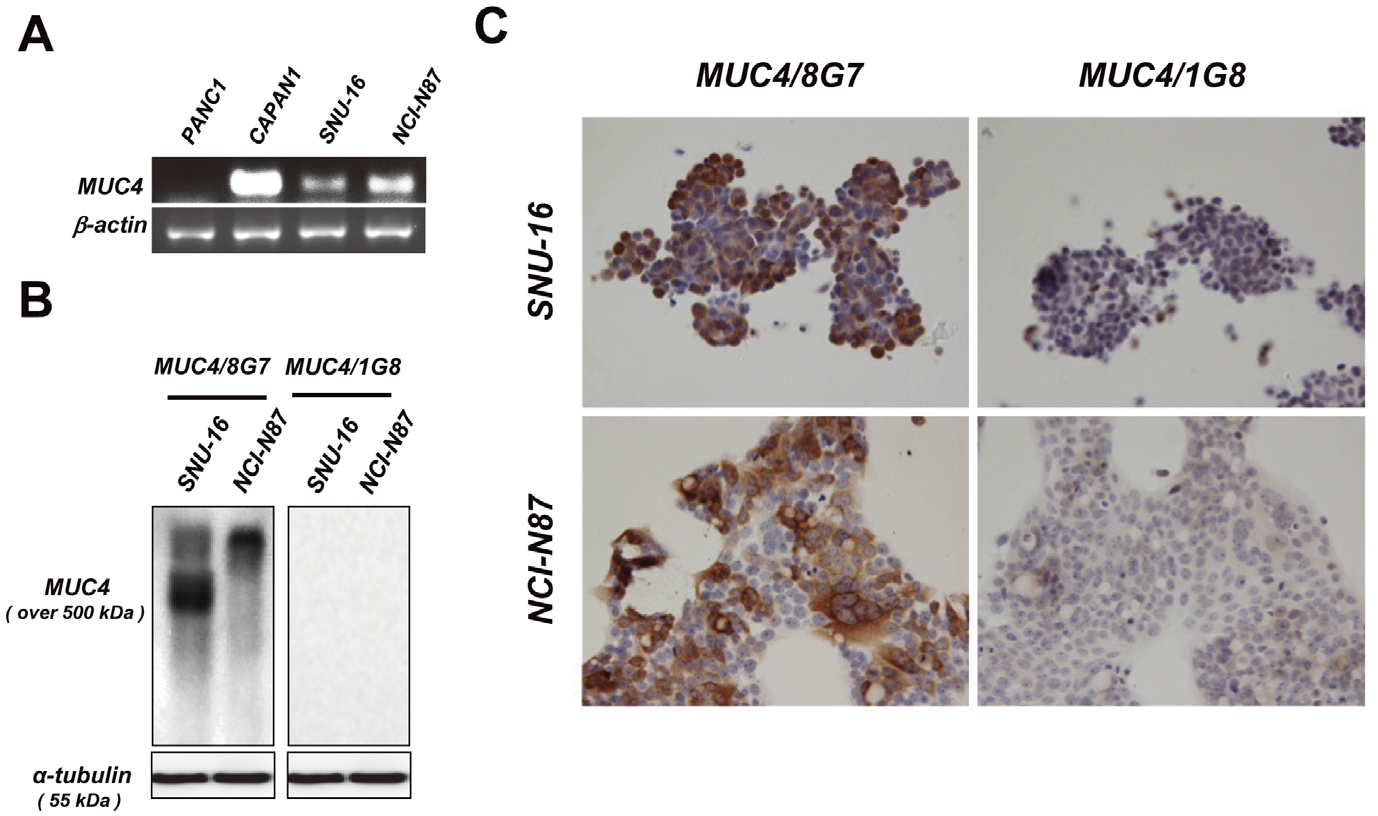
The difference in antibody specificity between anti-human MUC4 monoclonal antibodies (MAbs), 8G7 and 1G8. A: MUC4 mRNA was detected in the two gastric cancer cell lines, SNU-16 and NCI-N87. PANC1 and CAPAN1 cells were used as a negative and positive control, respectively. B: Cell lysates of SNU-16 and NCI-N87 were immunoblotted and detected by the indicated antibodies, respectively. A-tubulin served as a loading control. C: Formalin-fixed SNU-16 and NCI-N87 cells were processed for immunocytochemistry using the MAbs, 8G7 and 1G8, respectively. Original magnification ×400.

MUC4 was first reported as tracheobronchial mucin [7] and is a membrane-associated mucin [8]. In our study series, the expression of MUC4 in intrahepatic cholangiocarcinoma, pancreatic ductal adenocarcinoma, extrahepatic bile duct carcinoma, lung adenocarcinoma, and oral squamous cell carcinoma was an independent factor for poor prognosis and is a useful marker to predict the outcome of the patients [5], [9], [10], [11], [12], [13]. Unfortunatly, there are few studies of the MUC4 expression profile in human gastric cancer. In the present study, we examined the expression profiles of MUC4 as well as MUC1 in early gastric cancer tissues, and found that MUC4 and MUC1 expression in the early gastric cancers would become poor prognostic factors by lymph vessel invasion, blood vessel invasion and lymph node metastasis.

**Figure 2:**
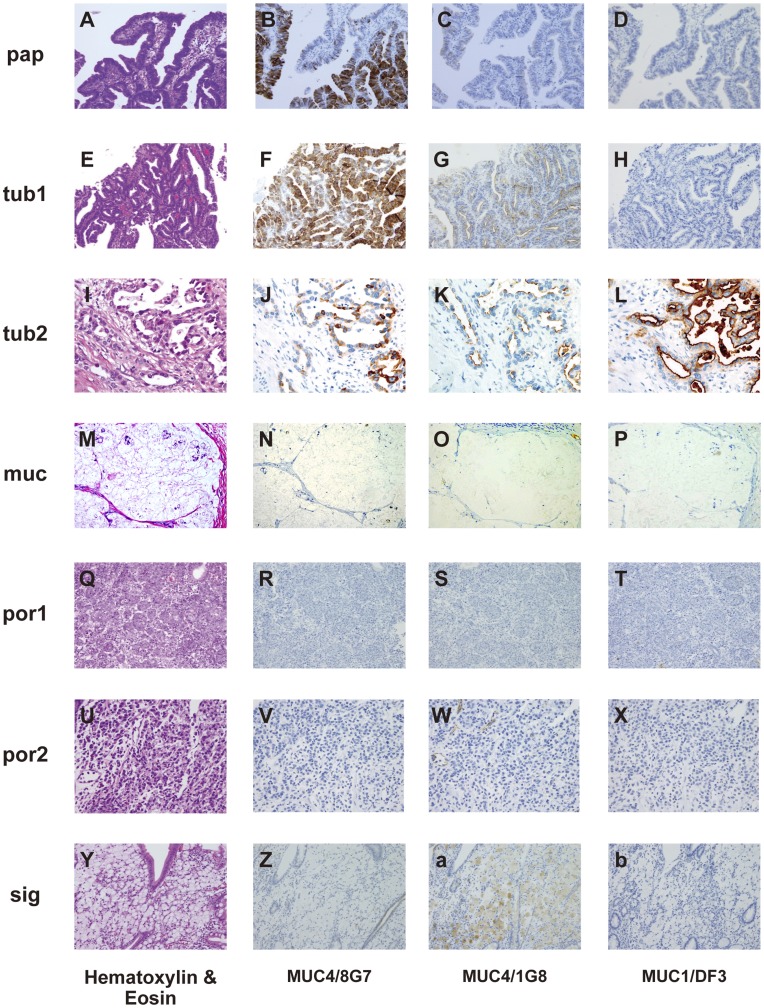
Expression patterns of MUC4/8G7, MUC4/1G8 and MUC1/DF3 in each histological type of gastric carcinoma. Hematoxylin-eosin (HE) (A), MUC4/8G7 (B), MUC4/1G8 (C) and MUC1/DF3 (D) in papillary adenocarcinoma (pap). HE (E), MUC4/8G7 (F), MUC4/1G8 (G) and MUC1/DF3 (H) in well differentiated tubular adenocarcinoma (tub1). HE (I), MUC4/8G7 (J), MUC4/1G8 (K) and MUC1/DF3 (L) in moderately differentiated tubular adenocarcinoma (tub2). HE (M), MUC4/8G7 (N), MUC4/1G8 (O) and MUC1/DF3 (P) in mucinous carcinomas (muc). HE (Q), MUC4/8G7 (R), MUC4/1G8 (S) and MUC1/DF3 (T) in solid type poorly differentiated adenocarcinoma (por1). HE (U), MUC4/8G7 (V), MUC4/1G8 (W) and MUC1/DF3 (X) in non-solid type poorly differentiated adenocarcinoma (por2). HE (Y), MUC4/8G7 (Z), MUC4/1G8 (a) and MUC1/DF3 (b) in signet-ring cell carcinoma (sig). MUC4/8G7 was expressed in the cytoplasm of pap (B), tub1 (F) and tub2 (J), but not in muc (N), por1 (R), por2 (V) nor sig (Z). MUC4/1G8 was expressed mainly at the cell apexes of pap (C), tub1 (G) and tub2 (K), but not in muc (O), por1 (S) nor por2 (W). MUC4/1G8 expression was seen in the intracytoplasmic mucin substance of sig (a). MUC1/DF3 was expressed mainly at the cell apexes tub2 (L), but not expressed in the cases shown in this figure (D, H, P, T, X and b). Original magnification ×200 (A–H, M–T), ×400 (I–L, U–b).

As anti-MUC4 monoclonal antibodies (MAbs), 8G7 and 1G8, are known to detect different sites of MUC4 molecule. The MAb 8G7 recognizes a tandem repeat sequence (STGDTTPLPVTDTSSV) of the human MUC4α subunit [14]. The MAb 1G8 is raised against the rat sequence (rat ASGP-2), and recognizes an epitope on the rat ASGP-2 subunit, which corresponds to the human MUC4β subunit, and shows a cross reactivity with human samples [15]. Thus, a special attention was paid to the comparison of two anti-MUC4 MAbs by Western blotting and IHC of two gastric cancer cell lines, before the IHC study of human gastric cancer tissues. Moreover, since there is controversy regarding the prognostic significance of these anti-MUC4 MAbs, a literature review of MUC4 expression in various cancers was also performed.

**Figure 3:**
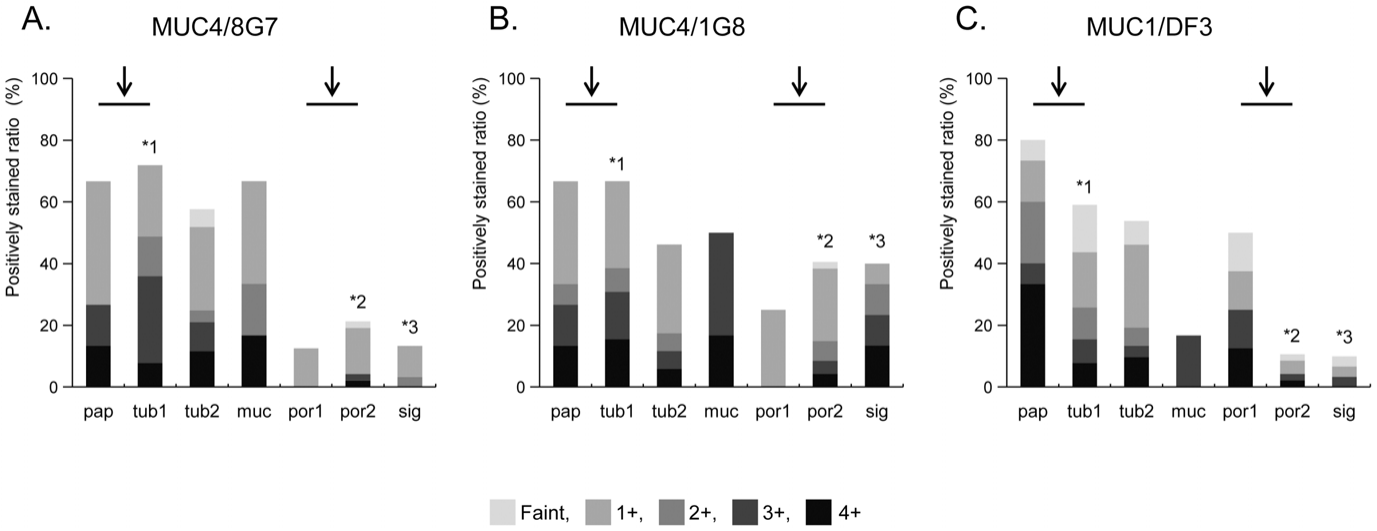
Semiquantitative evaluation of mucin expression in gastric carcinoma for each histological type (negative, none of the carcinoma cells stained; faint, >0% to <5% of carcinoma cells stained; 1+, ≥5% to <25%; 2+, ≥25% to <50%; 3+, ≥50% to <75%; and 4+: ≥75% stained. The detailed number and percentage of positively stained neoplastic cells using the scoring system were summarized in Table S1. MUC4/8G7, MUC4/1G8 and MUC1/DF3 expressions were were significantly higher in the well differentiated types (pap+tub1) than in the poorly differentiated type (por1+por2) (*P*<0.0001, *P* = 0.0021 and *P*<0.0001, respectively) (arrows). In tub1, expression rates of MUC4/8G7 and MUC4/1G8 were significantly higher than that of MUC1/DF3 (*P* = 0.0106 and *P* = 0.039, respectively) (*1). In por2, the expression rate of MUC4/1G8 was significantly higher than that of MUC4/8G7 (*P* = 0.0286) or that of MUC1/DF3 (*P* = 0.0005) (*2). In sig, the expression rate of MUC4/1G8 was significantly higher than that of MUC4/8G7 (*P* = 0.0158) or that of MUC1/DF3 (sig, *P* = 0.0019) (*3). In the other histolgical types (pap, tub2, muc and por1), there was no significant difference in the expression rates among MUC4/8G7, MUC4/1G8 and MUC1/DF3.

## Materials and Methods

### Patients and Tissue Samples

Gastrectomy specimens of 104 early gastric cancers, which show submucosal invasion, pT1b2, with or without lymph node metastasis, were retrieved from the file between 1994 and 2008 of the Kagoshima-shi Medical Association Hospital. The mean age of the patients was 65.7 (S.D., 9.8; range, 39–92 years; median age, 66 years); 64 cases were male, and 40 cases were female. This Study was conducted in accordance with the guiding principles of the Declaration of Helsinki, and approved by the Ethics Committee for Kagoshima-shi Medical Association Hospital (KMAH 2011-02-02). Informed, written consent was obtained from all patients. In the cases with more than two histological types mixed in one lesion, each histological pattern was evaluated independently, according to the Japanese Classification of Gastric Carcinoma (JCGC) [16].

**Table 1:** Relationship between expression of MUC4 and MUC1 and lymphatic invasion (ly), venous invasion (v) or lymph node metastasis (N).

	ly	v	N
MUC4/8G7 expression	r = 0.304	r = 0.280	r = 0.184
	P = 0.033	P = 0.083	P = 0.544
MUC4/1G8 expression	r = 0.395	r = 0.232	r = 0.296
	P = 0.001	P = 0.205	P = 0.045
MUC1/DF3 expression	r = 0.357	r = 0.377	r = 0.282
	P = 0.032	P = 0.024	P = 0.288

Spearman's rank-correlation coefficient.

### Evaluation of Monoclonal Antibodies for MUC4

#### Cells and culture conditions

Human gastric cancer cell lines (SNU-16 and NCI-N87) and pancreatic cancer cell lines (PANC1 and CAPAN1) were purchased from the American Type Culture Collection (Manassas, VA). Both gastric cancer cells were maintained in RPMI-1640 (Sigma-Aldrich, St Louis, MO); PANC1 cells were maintained in DMEM (Sigma-Aldrich); Capan1 cells were maintained in DMEM/F-12 (Sigma-Aldrich). All media were supplemented with 10% fetal bovine serum (GIBCO, Breda, The Netherlands) and 100 U/mL penicillin/100 µg/mL streptomycin (Sigma-Aldrich). All cells were incubated in 5% CO2 at 37°C and maintained at sub-confluent levels.

**Table 2:** Correlation among MUC4/8G7, MUC4/1G8 and MUC1/DF3.

Comparison		Correlation coefficient	P value
MUC4/8G7	MUC4/1G8	r = 0.486	P<0.0001
MUC4/8G7	MUC1/DF3	r = 0.267	P = 0.202
MUC4/1G8	MUC1/DF3	r = 0.245	P = 0.269

Spearman's rank-correlation coefficient.

#### RNA extraction and RT-PCR

Total RNA was extracted from the cells using the RNeasy mini kit (Qiagen, Hilden, Germany) and quantified by NanoDrop ND-1000 spectrophotometer. The obtained mRNA (2ug) was reverse transcribed to cDNA with the High Capacity RNA to cDNA kit (Applied Biosystems, Foster City, CA). The following primers were designed for the subsequent PCR: MUC4, 5′- TGGGACGATGCTGACTTCTC-3′, 5′-CCCCGTTGTTTGTCATCTTTC-3′; ACTB, 5′-CTCTTCCAGCCTTCCTTCCTG-3′, 5′-GAAGCATTTGCGGTGGACGAT-3′. PCR was performed with the AmpliTaq Gold Fast PCR Master Mix (Applied Biosystems) following the manufacturer’s protocol.

**Table 3:** Clinicopathological studies using anti-MUC4 monoclonal antibodies, 8G7 and 1G8.

Organ	Carcinoma type	Used Antibody	Correlation of MUC4 expression with outcome	Reference	Ref. No
Oral cavity	Squamous cell carcinoma	8G7	Poor	Hamada (2012)	[9]
Upper aerodigestive tract	Squamous cell carcinoma	1G8	Better	Weed (2004)	[28]
Salivary gland	Mucoepidermoid carcinoma	1G8	Better	Weed (2004)	[27]
Salivary gland	Mucoepidermoid carcinoma	1G8	No correlation	Handra-Luca (2005)	[20]
Salivary gland	Mucoepidermoid carcinoma	Rabbit polyclonal(Gut 2000 47:349)	Better	Alos (2005)	[19]
Thyroid	Papillary carcinoma	1G8	No expression of MUC4/1G8	Baek (2007)	[25]
Thyroid	Papillary carcinoma	1G8	Correlation with small tumorsize and microcarcinoma subtype,No comment for outcome	Nam (2011)	[23]
Lung	Small sized adenocarcinoma	8G7	Poor	Tsutsumida (2007)	[13]
Lung	Non–small cell lung carcinoma	1G8 (Zymed)	Better	Kwon (2007)	[29]
Lung	Non–small cell lung carcinoma(NSCLC)	1G8	Better	Jeon (2010)	[30]
Breast	Adenocarcinoma	1G8	No correlation	Rakha (2005)	[21]
Stomach	Adenocarcinoma	8G7	No association with tumortype, stage or with thedegree of differetiation, Nocomment for outcome	Senapati (2008)	[18]
Bile duct	Intrahepatic cholangiocarcinoma-mass forming type	8G7	Poor	Shibahara (2004)	[10]
Bile duct	Extrahepatic bile duct carcinoma	8G7	Poor	Tamada (2006)	[11]
Pancreas	Invasive ductal carcinoma	8G7	Poor	Saitou (2005)	[12]
Pancreas	Pancreatobiliary adenocarcinomas	1G8	Poor	Westgaard (2009)	[31]
Colon	Colorectal adenocarcinoma	8G7	Poor	Shanmugam (2010)	[26]
Ovary	Serous, mucinous, endometrioid andclear cell carcinoma	8G7	No correlation	Chauhan (2006)	[22]
Prostate	Prostate cancer	8G7	Down regulation in prostate cancer tissues, No comment for outcome	Singh (2006)	[24]

#### Protein extraction and western blotting

Total cell lysates were prepared using RIPA buffer containing protease inhibitor cocktail (Nacalai Tesque, Kyoto, Japan). The protein concentration was measured by the BCA assay (Thermo Scientific, Rockford, IL). An equal amount of protein lysate was resolved on 2% agarose gel containing SDS and passively transferred onto PVDF membrane overnight at room temperature. Membranes were blocked with 1% skim milk/PBST over 2 hours and subjected to the standard immunodetection procedure using specific primary antibodies. The primary antibodies are as follows: anti-human MUC4 MAb 8G7 (1∶1000, generated by Dr. Surinder K. Batra, University of Nebraska Medical Center, Omaha, NE) and 1G8 (1∶1000, purchased from Invitrogen, Camarillo, CA), and anti-human a-tubulin MAb DM1A (1∶2,000, Sigma-Aldrich).

#### Immunocytochemistry for cultured cells

For MUC4 staining in cultured cells, cells were seeded in 8-chamber slides (Becton Dickinson and Company, Franklin lakes, NJ) and incubated for overnight. Cells were fixed with 3.7% formaldehyde for 10min at room temperature and stained with MAb 8G7 (1∶24,000) and MAb 1G8 (1∶4,000) overnight at 4°C, respectively. Signal detection was performed by an immunoperoxidase method using a Vectastain Elite ABC kit (Vector Laboratories, Inc., Burlingame, CA) according to the manufacturer’s instructions.

### Immunohistochemistry for Human Tissues

IHC for human gastric carcinomas was done by using the following antibodies in the maximum cut sections in each tumor. MUC4 was detected by two MAbs, 8G7 and 1G8. For the comparative study, MUC1 expression was also examined by MAb DF3 (mouse IgG, TFB, Tokyo, Japan). IHC was performed by the immunoperoxidase method as follows. Antigen retrieval was performed using CC1 antigen retrieval buffer (pH8.5 EDTA, 10037°C, 30 min., Ventana Medical Systems, Tucson, AZ) for all sections. Following incubation with the primary antibodies (MAb MUC4/8G7 diluted 1∶3000, 37°C, 32 min.; MAb MUC4/1G8 diluted 1∶500, 37°C, 24 min.; MAb MUC1/DF3 diluted 1∶50, 37°C, 32 min.) in phosphate buffered saline pH 7.4 (PBS) with 1% bovine serum albumin, sections were stained on a Benchmark XT automated slide stainer using a diaminobenzidine detection kit (ultraView DAB, Ventana Medical Systems). For simplicity, MUC4/8G7, MUC4/1G8 and MUC1/DF3 are used to indicate the mucin antigens detected by each antibody.

#### Scoring of the results and statistical analysis

Four blinded investigators (Y.T., M.H., M.G. and S.Y.) evaluated the IHC staining data independently. When the evaluation differed among the four, a final decision was made by consensus. The results were evaluated based on the percentage of positively stained carcinoma cells, and the percentage data were categorized into six grades using the following scoring system: negative, none of the carcinoma cells stained; faint, <5% of carcinoma cells stained; 1+, ≥5% to <25%; 2+, ≥25% to <50%; 3+, ≥50% to <75%; and 4+: ≥75% stained. Cases with ≥5% of carcinoma cells stained were considered positive. Statistical analysis was performed by nonparametric methods using EXCEL-Statistics ver.3 software generated by Hisae Yanai, Faculty of Science, Saitama University (OMS Publishing, Japan).

Survival of the patients was compared between the group with positive MUC4/8G7, MUC4/1G8 or MUC1/DF3 expression and the group with negative expression according the Kaplan-Meier method, and differences between the survival curves were tested using the log-rank test. A probability of P<0.05 was considered statistically significant.

## Results

### Evaluation of Two Monoclonal Antibodies for MUC4

To investigate the difference in antibody specificity between 8G7 and 1G8, we carried out RT-PCR, Western blotting and IHC analysis using two gastric cancer cell lines, SNU-16 and NCI-N87 cells. The MUC4 mRNA was detected in the two gastric cancer cell lines (Fig. 1A). Consistent with the previous report [14], our data showed that 8G7 recognized a very high molecular weight protein (over 500 kD, which was the expected size for native MUC4). On the contrary, 1G8 does not show any immunoreactive bands (Fig. 1B). The same result was observed in the IHC analysis (Fig. 1C).

### Immunohistochemical Staining of Gastrectomy Specimens

#### Immunohistochemical staining of non-neoplastic gastric mucosa

In the non-neoplastic mucosa of the cases with gastric cancer, MUC4/8G7 was expressed sometimes in the cytoplasm of surface mucous epithelium, and frequently but weakly in the cytoplasm of fundic and pyloric glands (Figure S1 A and D). MUC4/1G8 was frequently expressed in the cell apex and cytoplasm of the surface mucous epithelium, and frequently but weakly in the cytoplasm of fundic and pyloric glands (Figure S1 B and E), and was seen constantly at the vascular endothelium. MUC1/DF3 was sometimes expressed in the surface mucous epithelium, and always in the fundic glands (particularly intensely at the cell apexes), but not in the pyloric glands (Figure S1 C and F).

#### Immunohistochemical staining of gastric adenocarcinoma

We examined gastrectomy specimens of 104 early gastric cancers (pT1b2), since we wished to avoid the major degenerative changes that are frequently seen in advanced cancer tissues, and to adjust the stage for the accurate comparison between IHC findings and the clinicopathologic factors.

When more than two histological types were mixed in one lesion in the gastrectomy specimens of 104 early gastric cancers, each histological pattern was evaluated independently, according to the JCGC [16]. Therefore, in the 104 gastrectomy specimens, we could evaluate 197 carcinoma foci of various histological types in total.

Among the 197 lesions, there were 15 lesions of papillary adenocarcinoma (pap) (Figs. 2A–D), 39 of well differentiated tubular adenocarcinoma (tub1) (Figs. 2E–H), 52 of moderately differentiated tubular adenocarcinoma (tub2) (Figs. 2I–L), 6 of mucinous carcinomas (muc) (Figs. 2M–P), 8 of solid type poorly differentiated adenocarcinoma (por1) (Figs. 2Q–T), 47 of non-solid type poorly differentiated adenocarcinoma (por2) (Figs. 2U–X) and 30 of signet-ring cell carcinoma (sig) (Figs. 2Y-b), based on the context of common histological classification of gastric cancer in JCGC [16]. According to the context in the WHO classification of tumours of the stomach [17] as well as that in JCGC [16], pap and tub1 were classified into “well-differentiated adenocarcinoma”, and por1 and por2 were classified into “poorly-differentiated adenocarcinoma”. The data of the expression rate of MUC4/8G7, MUC4/1G8 and MUC1/DF3 were summarized in Figure 3. The detailed number and percentage of positively stained neoplastic cells using the scoring system were summarized in Table S1.

#### Expression profile of MUC4/8G7

Among the 197 adenocarcinoma lesions, MUC4/8G7 was expressed in 83 lesions (42%). MUC4/8G7 showed a significantly higher rate of the positive expression (≥5% of carcinoma cells stained) in well differentiated types (pap+tub1: 70%, 38/54) than that in poorly differentiated types (por1+por2: 18%, 10/55) (*P*<0.0001) (Fig. 3A, arrows). MUC4/8G7 was expressed mainly in the cytoplasm of the neoplastic cells of pap (Fig. 2B), tub1 (Fig. 2F) and tub2 (Fig. 2J), in the cases with positive expression.

#### Expression profile of MUC4/1G8

Among the 197 adenocarcinoma lesions, MUC4/1G8 was expressed in 95 lesions (48%). MUC4/1G8 showed significantly higher rates of the positive expression in well differentiated types (pap+tub1: 67%, 36/54) than that in poorly differentiated types (por1+por2: 36%, 20/55) (*P* = 0.0021) (Fig. 3B, arrows). MUC4/1G8 was expressed mainly at the cell apexes of pap (Fig. 2C), tub1 (Figs. 2G) and tub2 (Fig. 2K), or in the intracytoplasmic mucin substance of sig (Fig. 2a), in the cases with positive expression.

#### Expression profile of MUC1/DF3

Among the 197 adenocarcinoma lesions, MUC1/DF3 was expressed in 62 lesions (31%). MUC1/DF3 showed significantly higher rates of the positive expression in well differentiated types (pap+tub1: 52%, 28/54) than that in poorly differentiated types (por1+por2: 13%, 7/55) (*P*<0.0001) (Fig. 3C, arrows). MUC1/DF3 was expressed mainly at the cell apexes of pap, tub1 and tub2 (Fig. 2L), in the cases with positive expression.

#### Comparison of mucin expression in each histologic type

In tub1, expression rates of MUC4/8G7 and MUC4/1G8 were significantly higher than that of MUC1/DF3 (*P* = 0.0106 and *P* = 0.039, respectively) (Fig. 3, *1). In por2, the expression rate of MUC4/1G8 was significantly higher than that of MUC4/8G7 (*P* = 0.0286) or that of MUC1/DF3 (*P* = 0.0005) (Fig. 3, *2). In sig, expression rate of MUC4/1G8 was significantly higher than that of MUC4/8G7 (*P* = 0.0158) or that of MUC1/DF3 (sig, *P* = 0.0019) (Fig. 3, *3). In the other histolgical types (pap, tub2, muc and por1), there was no significant difference in the expression rates among MUC4/8G7, MUC4/1G8 and MUC1/DF3 (Fig. 3).

#### Relationship between MUC4 or MUC1 expression and lymph vessel invasion, blood vessel invasion and lymph node metastasis

Semiquantitative evaluation of lymphatic invasion (ly), venous invasion (v) and lymph node metastasis status (N) is defined in the JCGC [16]. Lymphatic invasion (ly) was evaluated as follows; ly0, no lymphatic invasion; ly1, minimal lymphatic invasion; ly2, moderate lymphatic invasion; and ly3, marked lymphatic invasion. For the venous invasion (v), similar evaluation (v0 to v3) was done using elastic staining (Victoria-Blue) which was added to hematoxylin-eosin staining. The total number of lymph nodes and the number of involved lymph nodes at each nodal station (N) were recorded as follows; N0, no regional lymph node metastasis; N1, metastasis in 1–2 regional lymph nodes; and N2, metastasis in 3–6 regional lymph nodes. Regional lymph node metastasis was observed in 55 patients (N1, 41 cases; N2, 14 cases). There was no significant correlation between the histological types and lymphatic invasion, venous invasion or lymph node metastasis status.

In each case, the highest score of the six IHC grades (negative, faint, 1+, 2+, 3+ or 4+) in the various histological types was counted as the IHC score in each individual, e.g. “IHC score 4+” for a case with [tub1 score, 3+; tub2 score, 4+]. We evaluated the correlation between the IHC score and the ly, v and N factors in each patient. As shown in Table 1, the MUC4/8G7 expression was related with lymphatic invasion (r = 0.304, *P* = 0.033). The MUC4/1G8 expression was related with lymphatic invasion (r = 0.395, *P* = 0.001) and lymph node metastasis (r = 0.296, *P* = 0.045). The MUC1/DF3 expression was related with lymphatic invasion (r = 0.357, *P* = 0.032) and venous invasion (r = 0.377, *P* = 0.024).

Furthermore, we examined the correlation among the IHC scores of MUC4/8G7, MUC4/1G8 and MUC1/DF3. As shown in Table 2, there was a correlation between MUC4/8G7 score and MUC4/1G8 score (r = 0.486, *P*<0.0001). In contrast, there was no correlation between MUC4/8G7 score and MUC1/DF3 score (r = 0.267, *P* = 0.202), and there was no correlation between MUC4/1G8 score and MUC1/DF3 score (r = 0.245, *P* = 0.269).

#### Relationship between MUC4 or MUC1 expression and survival

Among the 104 patients, follow up data was obtained for 87 patients. Median follow up period was 47.5 months (range, 0–193 months). In the 87 patients, one patient died of the gastric carcinoma 39 months after surgery, one patient showed liver metastasis but survived for 78 months, and two patients died of other diseases. In 85 patients excluding two patients died of other diseases, Kaplan-Meier estimate was tested using the log-rank test. Between the positive group and negative group (MUC4/8G7+ vs −, MUC4/1G8+ vs − or MUC1/DF3+ vs −), log-rank test of overall survival (MUC4/8G7, P = 0.27; MUC4/1G8, P = 0.37; MUC1/DF3, P = 0.22) and progression-free survival (MUC4/8G7, P = 0.85; MUC4/1G8, P = 0.23; MUC1/DF3, P = 0.83) showed no significant differences. Further analysis of 42 patients survived for more than 5 years also showed no significant differences.

## Discussion

Recently, we have reported that the expression of MUC4 is an independent poor prognostic factor of pancreatobiliary adenocarcinomas [10], [11], [12] as well as lung adenocarcinoma [13] and oral squamous cell carcinoma [9]. MUC1 has also been reported to be a poor prognostic factor in various human neoplasms [4], [5]. Our previous study in gastric cancers, including both early cancers and advanced cancers demonstrated that MUC1 is a useful prognostic factor for poor outcome in the patients [6]. In the present study, the relationship between mucin expression and the patient’s outcome cannot be evaluated, because the gastric cancers are in the early stage at pT1b2 and most of the patients have had a favorable outcome. Nevertheless, the following results were obtained: (1) The MUC4/8G7, MUC4/1G8 and MUC1/DF3 expressions were related with lymphatic invasion. (2) The MUC4/1G8 expression was related with lymph node metastasis. (3) The MU1/DF3 expression was related with venous invasion. In Japan, ESD is the first choice treatment for early gastric cancers [3]. Examination of MUC4 as well as MUC1 in the ESD specimens may clarify whether the additional surgery, including lymph node dissection or frequent follow-up for the metastasis are necessary.

Our previous studies demonstrated that there was no siginificant correlation between MUC4 expression and MUC1 expression [10], [11], [12], [13]. Also in the present study of the gastric cancers in the early stage, there was no siginificant correlation between expression of MUC4 and MUC1. Both MUC4 and MUC1 expression in the gastric cancers may be related with the poor prognostic factors, such as lymphatic invasion, venous invasion and lymph node metastasis, by means of different mechanism.

In the previous study of gastric cancers using MAb 8G7, Senapati et al. demonstrated that MUC4/8G7 expression was not associated with tumor type, stage or with the degree of differentiation [18]. Interestingly, their results showed an 42% expression rate in the stage I cancers (n = 19), which is in accordance with our data (MUC4/8G7: 42% and MUC4/1G8: 48%) in the present study examining stage I cancers (n = 104). However, our study revealed that both MUC4/8G7 and MUC4/1G8 expressions were different among the histological types, and were significantly higher in the well differentiated types than in the poorly differentiated type. MUC1/DF3 expression was also significantly higher in the well differentiated types than in the poorly differentiated type. We reported that MUC1 expression was high in the well differentiated adenocarcinoma in gastric cancers including advanced cancers, and the high MUC1 expression may affect the survival of patients with well differentiated adenocarcinoma of stomach [6]. The high expression of MUC4 in the well differentiated adenocarcinoma also may affect the survival of patients with gastric cancer. In our previous study [6], the rate of high expression of MUC1/DF3 was significantly higher in the advanced gastric cancers than that in the early gastric cancers. The relationship of MUC4 expression with the invasion of gastric cancers would be an interesting area of study.

There is controversy regarding the prognostic significance of MUC4/8G7 and MUC4/1G8 expression. Thus, we have reviewed 19 articles of MUC4 IHC study applied for various human cancer tissues (Table 3). The significance of MUC4/8G7 and MUC4/1G8 could not be evaluated in 8 of the 19 studies. One study using polyclonal anti-MIUC4 antibody reported that MUC4 expression is related to a fovorabel outcome [19], three studies show no correlation between MUC4 expression and prognosis [20], [21], [22], the other three studies did not have any comments on the correlation between MUC4 expression and prognosis [18], [23], [24], and the remaining one study of thyroid cancer reported no MUC4 expression in the cancer [25]. On the other hand, in the other 11 articles, there was an apparent difference of the prognostic significance between MUC4/8G7 expression and MUC4/1G8 expression. Most studies using 8G7, which was generated against human MUC4, MUC4/8G7 expression is related to aggressive tumor behavior or a poor outcome in human carcinomas [9], [10], [11], [12], [13], [26]. In contrast, most studies using 1G8, which was raised against rat ASGP-2, described that MUC4/1G8 expression is related to a favorable outcome [27], [28], [29], [30], although one study of pancreatic adenocarcinoma described that MUC4/1G8 expression is related to poor survival [31]. This clear difference raises the question of whether 8G7 and 1G8 have essentially different characters. The MAb 1G8 was raised using rat Muc4 epitope [15]. Human MUC4 and rat Muc4 shows more than 60% peptide sequence similarity [32], but they are not identical. It is noteworthy that IHC using MAb 1G8 always shows positive staining in the vascular endothelium, which is somewhat unusual as the expression of MUC4 which is one of the members of mucins.

Thus, we evaluated the specificity of the MAb 8G7 and MAb 1G8 by Western blotting and IHC of two gastric cancer cell lines. Our Western blotting analysis showed that MAb 8G7 recognized a very high molecular weight protein (over 500 kD, which was the expected size for native MUC4), whereas MAb 1G8 does not show any immunoreactive bands. The IHC analysis also showed MAb 8G7 positive staining but MAb 1G8 negative staining in the two gastric cancer cell lines. MUC4 mRNA was also expressed in the two gastric cancer cell lines in the present study, as shown in the previous study analyzing the pancreatic cancer cell lines by RT-PCR and northen blot analyses [33], [34]. Both MAb 8G7 and MAb 1G8 react with human gastric cancer tissues, although the locations of MUC4/8G7 and MUC4/1G8 expression showed a marked difference. In gastric cancer tissues, MUC4/8G7 was expressed mainly in the cytoplasm of the neoplastic cells of pap and tub, whereas MUC4/1G8 was expressed mainly at the cell apexes of pap and tub or intracytoplasmic mucin substance of sig. Since the cytoplasmic expression pattern of MUC4/8G7 is seen also in pancreatic adenocarcinoma, intrahepatic cholangiocarcinoma, extra hepatic bile duct carcinoma, lung adenocarcinoma and oral squamous cell carcinoma [9], [10], [11], [12], [13], the intracytoplasmic MUC4/8G7 expression pattern in gastric cancer tissues may be reasonable. In contrast, the linear expression pattern of MUC4/1G8 along with the cell apexes of gastric cancer tissues may reflect unknown functions or characteristics of the MUC4β subunit detected by MAb 1G8 raised against rat epitope [15], as the present study demonstrated that MUC4/1G8 expression were related to lymphatic invasion and lymph node metastasis that are poor prognostic factors even in the early gastric cancer. Particularly in por2 and sig, the expression rate of MUC4/1G8 was significantly higher than that of MUC4/8G7 or that of MUC1/DF3. In addition, there was a siginificant correlation between MUC4/8G7 expression and MUC4/1G8 expression in the patients examined. Thus, the IHC signal of MUC4/1G8 detected in the gastrectomy specimens may show a significant meaning of the epitope detected by MAb 1G8, although there was no reactivity of MUC4/1G8 expression in human gastric cancer cell lines (SNU-16 and NCI-N87). The epitope detected by MAb 1G8 is an area of interest for future study.

In conclusion, in the present study of early gastric cancers, MUC4/8G7, MUC4/1G8 and MUC1/DF3 expressions were observed mainly in well differentiated adenocarcinomas. The MUC4/8G7 expression was related with lymphatic invasion. The MUC4/1G8 expression was related with lymphatic invasion and lymph node metastasis. The MUC1/DF3 expression was related with lymphatic invasion and venous invasion. The examination of MUC4 and MUC1 expression in the gastric cancers would become a useful marker to predict poor prognostic factors related with vessel invasion, even in the early stage.

## Supporting Information

Figure S1 In the non-neoplastic mucosa of the cases with gastric cancer, MUC4/8G7 was expressed sometimes in the cytoplasm of surface mucous epithelium, and frequently but weakly in the cytoplasm of fundic and pyloric glands (A and D). MUC4/1G8 was frequently expressed in the cell apex and cytoplasm of the surface mucous epithelium, and frequently but weakly in the cytoplasm of fundic and pyloric glands (B and E), and was seen constantly at the vascular endothelium. MUC1/DF3 was sometimes expressed in the surface mucous epithelium, and always in the fundic glands (particularly intensely at the cell apexes), but not in the pyloric glands (C and F). Original magnification ×100 (A, B, C), ×400 (D, E, F).
(TIF)

Table S1 Detailed number and percentage of positively stained neoplastic cells using the scoring system.
(DOC)
