# Supplementary figures and images for: MUC4 and MUC1 Expression in Adenocarcinoma of the Stomach Correlates with Vessel Invasion and Lymph Node Metastasis: An Immunohistochemical Study of Early Gastric Cancer
Source: PLoS One. 2012 Nov 13;7(11):e49251. doi: 10.1371/journal.pone.0049251 (PMC3496698; doi:10.1371/journal.pone.0049251)

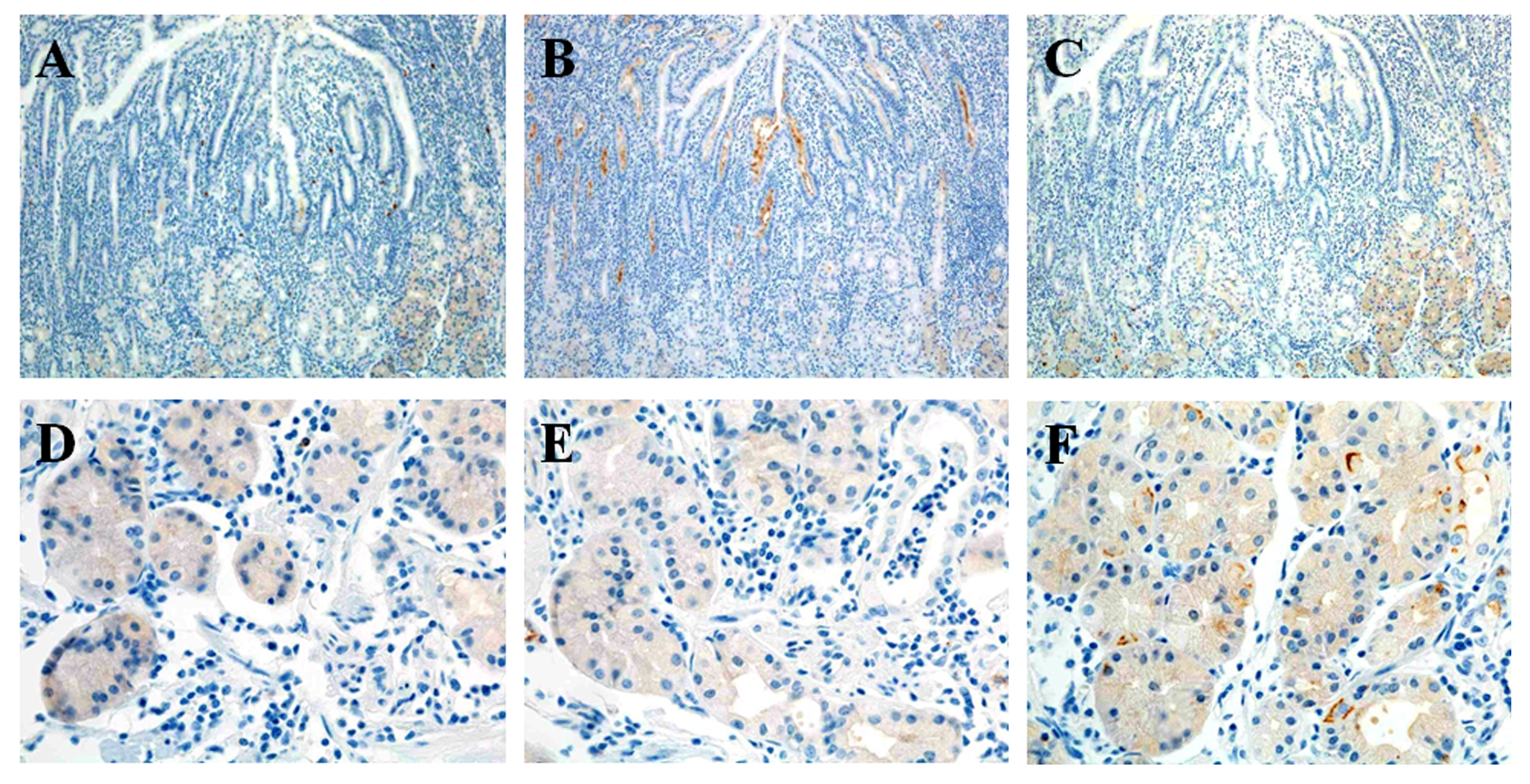

Supplement: Figure S1 — In the non-neoplastic mucosa of the cases with gastric cancer, MUC4/8G7 was expressed sometimes in the cytoplasm of surface mucous epithelium, and frequently but weakly in the cytoplasm of fundic and pyloric glands (A and D). MUC4/1G8 was frequently expressed in the cell apex and cytoplasm of the surface mucous epithelium, and frequently but weakly in the cytoplasm of fundic and pyloric glands (B and E), and was seen constantly at the vascular endothelium. MUC1/DF3 was sometimes expressed in the surface mucous epithelium, and always in the fundic glands (particularly intensely at the cell apexes), but not in the pyloric glands (C and F). Original magnification ×100 (A, B, C), ×400 (D, E, F). (TIF) [file pone.0049251.s001.tif]
