# Supplementary material for: MUC4 and MUC1 Expression in Adenocarcinoma of the Stomach Correlates with Vessel Invasion and Lymph Node Metastasis: An Immunohistochemical Study of Early Gastric Cancer
Source: PLoS One. 2012 Nov 13;7(11):e49251. doi: 10.1371/journal.pone.0049251 (PMC3496698; doi:10.1371/journal.pone.0049251)
Supplement: Table S1 — Detailed number and percentage of positively stained neoplastic cells using the scoring system. (DOC) [file pone.0049251.s002.doc]

| Table S1. Detailed number and percentage of positively stained neoplastic cells using the scoring system | | | | | | | |
| --- | --- | --- | --- | --- | --- | --- | --- |
| MUC4/8G7 | | | | | | | |
|  | pap | tub1 | tub2 | muc | por1 | por2 | sig |
| 0 | 5 (33.3%) | 11 (28.2%) | 22 (42.3%) | 2 (33.3%) | 7 (87.5%) | 37 (78.7%) | 26 (86.7%) |
| Faint | 0 (0.0%) | 0 (0.0%) | 3 (5.8%) | 0 (0.0%) | 0 (0.0%) | 1 (2.1%) | 0 (0.0%) |
| 1+ | 6 (40.0%) | 9 (23.1%) | 14 (26.9%) | 2 (33.3%) | 1 (12.5%) | 7 (14.9%) | 3 (10.0%) |
| 2+ | 0 (0.0%) | 5 (12.8%) | 2 (3.8%) | 1 (16.7%) | 0 (0.0%) | 0 (0.0%) | 1 (3.3%) |
| 3+ | 2 (13.3%) | 11 (28.2%) | 5 (9.6%) | 0 (0.0%) | 0 (0.0%) | 1 (2.1%) | 0 (0.0%) |
| 4+ | 2 (13.3%) | 3 (7.7%) | 6 (11.5%) | 1 (16.7%) | 0 (0.0%) | 1 (2.1%) | 0 (0.0%) |
| Total | 15 | 39 | 52 | 6 | 8 | 47 | 30 |
| MUC4/1G8 | | | | | | | |
|  | pap | tub1 | tub2 | muc | por1 | por2 | sig |
| 0 | 5 (33.3%) | 13 (33.3%) | 28 (53.8%) | 3 (50.0%) | 6 (75.0%) | 28 (59.6%) | 18 (60.0%) |
| Faint | 0 (0.0%) | 0 (0.0%) | 0 (0.0%) | 0 (0.0%) | 0 (0.0%) | 1 (2.1%) | 0 (0.0%) |
| 1+ | 5 (33.3%) | 11 (28.2%) | 15 (28.8%) | 0 (0.0%) | 2 (25.0%) | 11 (23.4%) | 2 (6.7%) |
| 2+ | 1 (6.7%) | 3 (7.7%) | 3 (5.8%) | 0 (0.0%) | 0 (0.0%) | 3 (6.4%) | 3 (10.0%) |
| 3+ | 2 (13.3%) | 6 (15.4%) | 3 (5.8%) | 2 (33.3%) | 0 (0.0%) | 2 (4.3%) | 3 (10.0%) |
| 4+ | 2 (13.3%) | 6 (15.4%) | 3 (5.8%) | 1 (16.7%) | 0 (0.0%) | 2 (4.3%) | 4 (13.3%) |
| Total | 15 | 39 | 52 | 6 | 8 | 47 | 30 |
| MUC1/DF3 | | | | | | | |
|  | pap | tub1 | tub2 | muc | por1 | por2 | sig |
| 0 | 3 (20.0%) | 16 (41.0%) | 24 (46.2%) | 5 (83.3%) | 4 (50.0%) | 42 (89.4%) | 27 (90.0%) |
| Faint | 1 (6.7%) | 6 (15.4%) | 4 (7.7%) | 0 (0.0%) | 1 (12.5%) | 1 (2.1%) | 1 (3.3%) |
| 1+ | 2 (13.3%) | 7 (17.9%) | 14 (26.9%) | 0 (0.0%) | 1 (12.5%) | 2 (4.3%) | 1 (3.3%) |
| 2+ | 3 (20.0%) | 4 (10.3%) | 3 (5.8%) | 0 (0.0%) | 0 (0.0%) | 0 (0.0%) | 0 (0.0%) |
| 3+ | 1 (6.7%) | 3 (7.7%) | 2 (3.8%) | 1 (16.7%) | 1 (12.5%) | 1 (2.1%) | 1 (3.3%) |
| 4+ | 5 (33.3%) | 3 (7.7%) | 5 (9.6%) | 0 (0.0%) | 1 (12.5%) | 1 (2.1%) | 0 (0.0%) |
| Total | 15 | 39 | 52 | 6 | 8 | 47 | 30 |
